# Applying artificial intelligence to longitudinal imaging analysis of vestibular schwannoma following radiosurgery

**DOI:** 10.1038/s41598-021-82665-8

**Published:** 2021-02-04

**Authors:** Cheng-chia Lee, Wei-Kai Lee, Chih-Chun Wu, Chia-Feng Lu, Huai-Che Yang, Yu-Wei Chen, Wen-Yuh Chung, Yong-Sin Hu, Hsiu-Mei Wu, Yu-Te Wu, Wan-Yuo Guo

**Affiliations:** 1 Department of Biomedical Imaging and Radiological Sciences, National Yang-Ming University, Taipei, Taiwan; 2 Department of Radiology, Taipei Veteran General Hospital, Taipei, Taiwan; 3 School of Medicine, National Yang-Ming University, Taipei, Taiwan; 4 Department of Neurosurgery, Neurological Institute, Taipei Veteran General Hospital, Taipei, Taiwan; 5 Institute of Biophotonics, National Yang-Ming University, Taipei, Taiwan; 6 Brain Research Center, National Yang-Ming University, Taipei, Taiwan

**Keywords:** Learning algorithms, Neurological disorders, CNS cancer, Cancer, Surgical oncology, Neuroscience, Medical research, Neurology, Oncology

## Abstract

Artificial intelligence (AI) has been applied with considerable success in the fields of radiology, pathology, and neurosurgery. It is expected that AI will soon be used to optimize strategies for the clinical management of patients based on intensive imaging follow-up. Our objective in this study was to establish an algorithm by which to automate the volumetric measurement of vestibular schwannoma (VS) using a series of parametric MR images following radiosurgery. Based on a sample of 861 consecutive patients who underwent Gamma Knife radiosurgery (GKRS) between 1993 and 2008, the proposed end-to-end deep-learning scheme with automated pre-processing pipeline was applied to a series of 1290 MR examinations (T1W+C, and T2W parametric MR images). All of which were performed under consistent imaging acquisition protocols. The relative volume difference (RVD) between AI-based volumetric measurements and clinical measurements performed by expert radiologists were + 1.74%, − 0.31%, − 0.44%, − 0.19%, − 0.01%, and + 0.26% at each follow-up time point, regardless of the state of the tumor (progressed, pseudo-progressed, or regressed). This study outlines an approach to the evaluation of treatment responses via novel volumetric measurement algorithm, and can be used longitudinally following GKRS for VS. The proposed deep learning AI scheme is applicable to longitudinal follow-up assessments following a variety of therapeutic interventions.

## Introduction

Advances in computing have opened the door to the development of artificial intelligence (AI) for a wide range of medical applications. AI has been applied to imaging analysis in radiology, pathology, and neurosurgery, and advanced AI systems incorporating the expertise of physicians and big data have achieved diagnostic accuracy exceeding 80%^1^. It is expected that AI will soon be used to optimize strategies for the clinical management of patients requiring intensive imaging follow-up, such as those who undergo radiation therapy for malignant glioma or lung cancer patients undergoing immunotherapy or targeted therapy^2,3^. This type of analysis requires the longitudinal analysis of medical images pertaining to multiple regions of interest.

Neurosurgery and radiation oncology are seen as a potential vanguard to guide the application of big-data analysis techniques in cancer research, quality assessment, and clinical care^4^. In the field of neuro-oncology, researchers analyzing the effects of radiation therapy must consider patient demographics, the specifics of the radiation treatment, imaging guidance techniques, and follow-up images generated over periods spanning a few days to several months or years. Taking an example from radiosurgery for vestibular schwannoma (VS), it originates from the schwann cell sheath of vestibulo-cochlear nerves and causes damage of vestibular function with a high risk of deafness and facial palsy by tumor progression or treatment. Gamma Knife radiosurgery (GKRS) is a safe and effective strategy to treat VSs with an over 90% long-term tumor control rate and a lower risk of treatment-related complications^5–9^. However, many VS patients exhibit tumor swelling for up to 1–2 years before notable shrinking occurs. Under these circumstances, long-term MR imaging follow-up and volume measurements are crucial to clinical decision-making.

Over the last three decades, researchers at our institute have been characterizing the anatomic details of target lesions following Gamma Knife radiosurgery (GKRS) based largely on the meticulous analysis of multi-parametric MR images (i.e., T1W, T1W+C, and T2W)^10^. We have found that the results of manual tumor contouring (even by experienced team members) tend to be subjective. Obtaining accurate assessments pertaining to the effects of GKRS treatment requires a reliable system by which to automate the segmentation and volume measurement of VS from follow-up MR images. Especially for patients with a VS underwent transient tumor growth after GKRS, the phenomenoma of pseudoprogression is usually occurs during 6–18 months and need meticulous volume measurements. Our objective in this study was to establish an algorithm by which to automate the volumetric measurement of VS using a series of parametric MR images. Wang and Shapey et al. have proposed a 2.5D U-Net^11,12^ and then employed additional spatial attention maps to demonstrate the feasibility of CNN modeling to segment VSs using anisotropic T1W+C and T2W MR images. Specifically, we trained a convolutional neural network (CNN) to identify tumor regions of inhomogeneous intensity to reduce the errors of volumetric measurement. The proposed AI scheme is applicable to longitudinal follow-up assessments following a variety of therapeutic interventions.

## Results

### Dual-pathway model for tumor delineation

**Figure 1:**
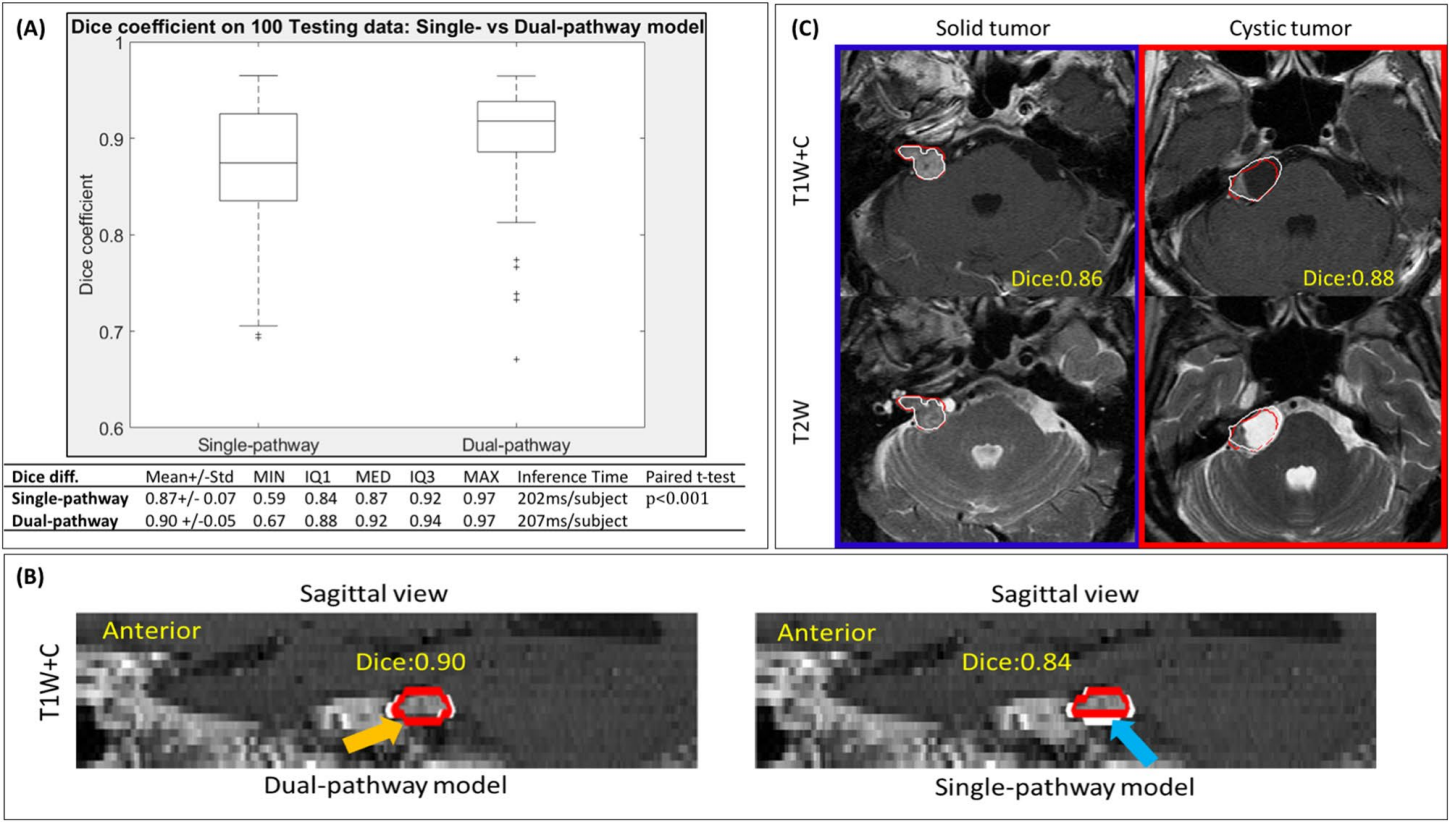
(**A**) Box plots showing dice scores for single-pathway model and dual-pathway model applied to 100 testing data sets (both models trained using T1W+C, and T2W images with Mean: average, Std: standard deviation, MIN: minimum, IQ1: 25th interquartile, IQ3: 75th interquartile, MED: median, MAX: maximum); (**B**) white ground-truth contour on left overlapping red contours predicted for a solid tumor using proposed dual-pathway model (dice score: 0.90); white ground-truth contour on right overlapping red contours predicted for same solid tumor using single-pathway model (dice score: 0.84); (**C**) upper and lower panels respectively display T1W+C and T2W images, where blue and red boxes respectively present testing results from cases of solid tumor and cystic tumor.

A total of 381 patients were recruited for analysis, the clinical details of which are summarized in Table 1. We first compared the tumor mask based on model segmentation with the results of manual delineation by experienced neurosurgeons (CC Lee, HC Yang, or WY Chung) and neuroradiologists (HM Wu, CC Wu, or WY Guo). When applied to 100 testing data, the proposed dual-pathway model significantly outperformed the single-pathway model (paired t test; p-value of < 0.001). The dice scores were as follows: 0.90 ± 0.05 (dual-pathway model) and 0.87 ± 0.07 (single-pathway model) (mean ± SD). Figure 1A lists the dice scores in terms of mean, median, standard deviation, maximal, minimal, and interquartile values. The case in Fig. 1B clearly illustrates the advantages of the dual-pathway model, with the following results: 0.84 (single-pathway model) and 0.90 (dual-pathway model).

**Table 1:** Demographic characteristics of 381 patients with vestibular schwannoma.

Characteristics	Value (n or median)	Range or percentage
Sex (male: female)	163:218	42.7%
Age (range), y/o	53.9	14.9–83.1
Laterality (left: right)	209:172	54.9%
Tumor volume (ml)	2.05	0.08–17.1
**Tumor characteristics**		
Solid	168	44.1%
Cystic	17	4.8%
Mixed	172	45.1%
**Neurological deficits**		
Hearing impairment	285	74.8%
Dizziness, tinnitus, imbalance	259	68.0%
Facial palsy	48	12.6%
Other CN deficits	46	12.1%
Long-tract sign (gait or weakness)	44	11.5%
Cerebellar sign	42	11.0%
**SRS parameters**		
Max dose (Gy)	21.0	15.4–24.6
Margin dose (Gy)	12.0	11.0–15.0
Isodose level (%)	57.0	50–85
Clinical follow-up	73.0	6.1–217
Image follow-up (in months)	71.3	5.6–217
**Image outcomes**		
Regression or stable	168	44.1%
Pseudo-progression	172	45.1%
Progression	17	4.8%

### Cystic VS and loss of central enhancement after GKRS

Cystic or mixed cystic tumors are not uncommon in patients with VS, and radiosurgery often leads to a loss of central enhancement during the follow-up period. Note that in either of these situations, most AI models are susceptible to volumetric error. It appears that most AI models are able to interpret contrast-enhanced lesions with a high degree of accuracy; however, they tend to underestimate the cystic component of the tumor due to a loss of enhancement. Finally, most AI models can be trained only for specific types of VS. The proposed dual-pathway model was designed to overcome these limitations by combing the signals from T2W, and T1W+C images. This makes it possible to measure the volume of a tumor with a high degree of precision, even when dealing with cystic tumors or a loss of central enhancement following radiation treatment. Figure 1C illustrates the advantages of the proposed dual-pathway model. Despite the fact that this tumor is largely composed of cystic fluid, it is well-covered and segmented, with a dice value of 0.88. In fact, the dice values obtained from solid, cyst, and mixed tumors using the proposed model are comparable.

### Longitudinal analysis of parametric MR images

**Figure 2:**
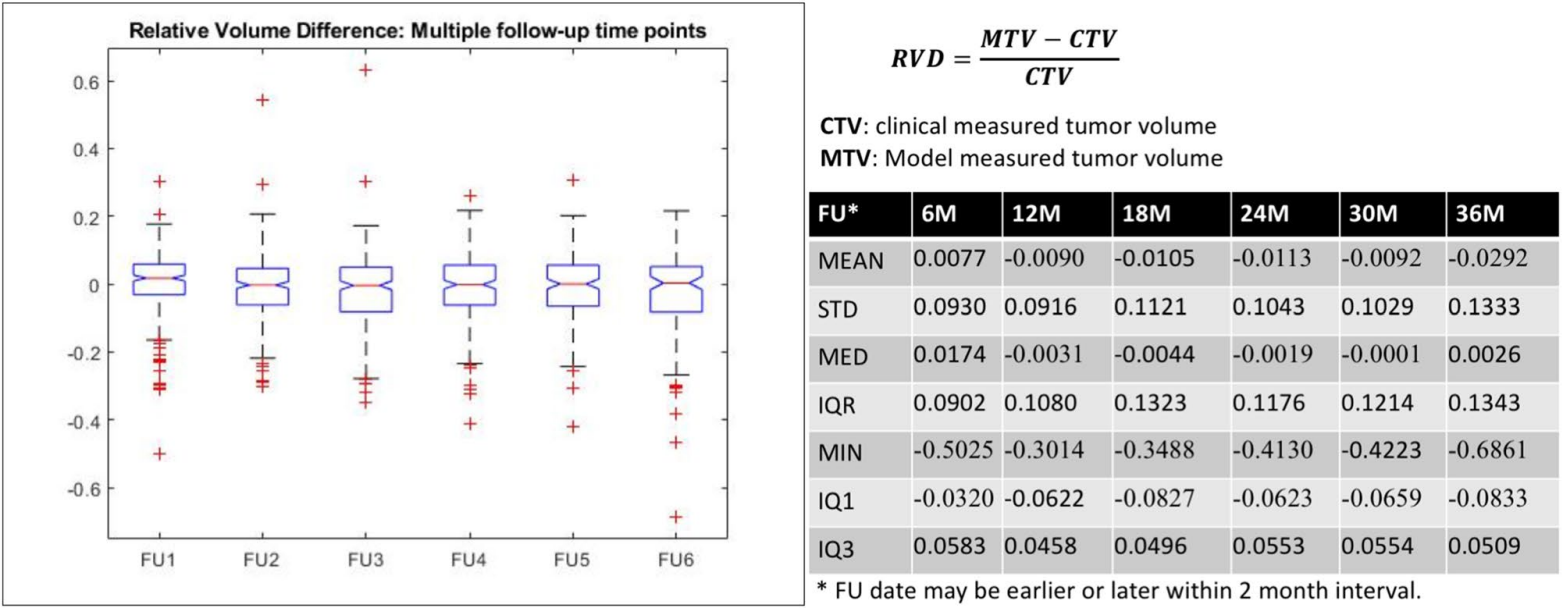
Longitudinal analysis of parametric MR images from 381 VS patients. Regardless of the state of the tumor (progressed, pseudo-progressed, or regressed), there were negligible differences between the predictions of the proposed AI model and clinical measurements obtained by expert radiologists. The median differences are presented here at each follow-up time point: + 1.74%, − 0.31%, − 0.44%, − 0.19%, − 0.01%, and + 0.26 (Mean: average, Std: standard deviation, MIN: minimum, IQ1: 25th interquartile, IQ3: 75th interquartile, MED: median, MAX: maximum).

The dual-pathway model was used for the longitudinal analysis of parametric MR images to assess changes in tumor volume, the results of which are presented in Fig. 2. Regardless of the state of the tumor (progressed, pseudo-progressed, or regressed), the RVDs between model-measured tumor volume (MTV) with the clinically-measured tumor volume (CTV) are minimal. The mean differences at the follow-up time points were + 0.77%, − 0.90%, − 1.05%, − 1.13%, − 0.92%, and − 2.92%. The median differences were + 1.74%, − 0.31%, − 0.44%, − 0.19%, − 0.01%, and + 0.26%. Note that the accuracy of the proposed AI model was roughly 99% at most follow-up time points.

### Case demonstration

**Figure 3:**
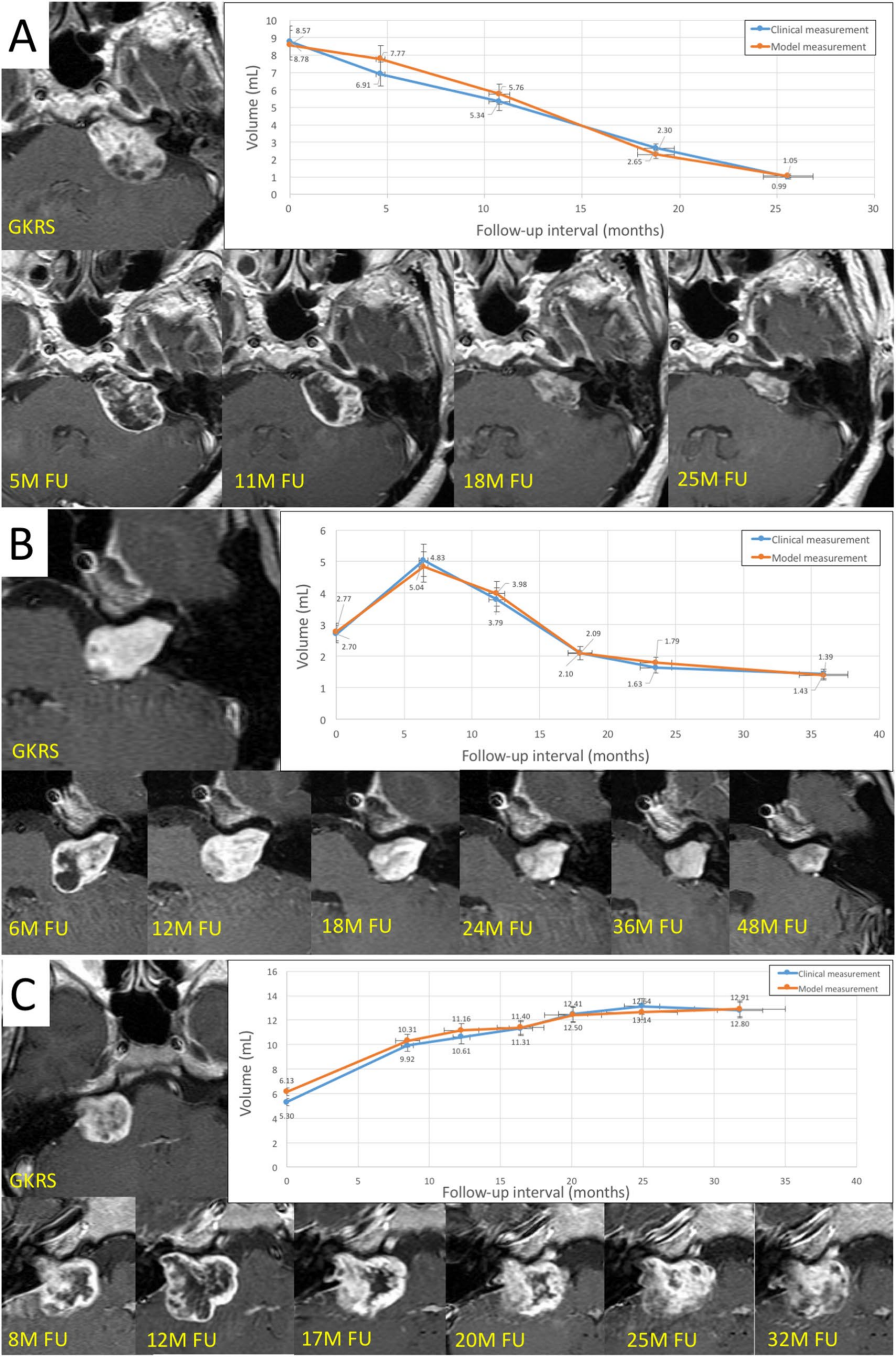
Case demonstration: (**A**) 46 y/o female VS patient who underwent GKRS and sequential MRI follow-up (follow-up MR images revealed positive response to radiation and ongoing tumor regression); (**B**) 53 y/o male VS patient who underwent GKRS and sequential MRI follow-up (follow-up MR images also showed positive response to radiation treatment with loss of MR imaging signal in center of tumor but transient enlargement of tumor); (**C**) 43-year-old female VS patient who underwent GKRS and sequential MRI follow-up (follow-up MR images obtained at 6 months after GKRS showed poor response to radiation treatment). A loss of central enhancement was observed in the early stage after GKRS; however, the tumor continued growing. At most follow-up time points, the differences between the predictions of AI models and clinical measurements were less than 10%. The trends of the tumor progression or regression predicted by the proposed AI model were remarkably accurate.

In the following, we present three case studies involving (1) VS with direct tumor regression, (2) pseudo-progression leading to tumor regression, and (3) tumor progression. The former two cases responded favorably to radiosurgery, i.e., tumor shrinkage without any adverse radiation effects. MTV and CTV results were similar, with an RVD difference of less than 10% at most follow-ups in all of three patients. (Fig. 3). The proposed AI model was highly accurate.

## Discussion

Regardless of the state of the tumor (progressed, pseudo-progressed, or regressed), the differences between the results of the proposed AI model and the clinical measurements obtained by expert radiologists were within the range deemed clinically acceptable, i.e., less than 1% at all time points other than the first (+ 1.74%). Note that this level of precision is far superior to the 10% measurement error that is normally deemed acceptable^13^.

Numerous researchers have addressed the issue of VS segmentation using MR images. Elizabeth et al.^14^ proposed a Bayesian partial volume segmentation scheme based on probability statistics. Deep-learning techniques, such as convolutional neural networks (CNNs), have also been developed for image recognition and semantic segmentation. These methods use convolution kernels to produce feature maps aimed at facilitating classification tasks. They have also been applied in medical imaging research^15^. Wang et al.^11,16^ proposed the 2.5D U-Net for VS segmentation, in which T2W MR images were used as input data and spatial attention maps were used to identify tumors from MR images with anisotropic resolution. Differences between the Wang study and current study in terms of through-plane resolution (1.5 mm vs. 3.0 mm), scanning parameters, and sample size (*N* = 46 vs *N* = 100) preclude the direct comparison of results. Nonetheless, the excellent results of their study (dice coefficient of 0.93 ± 0.04) demonstrate the feasibility of using CNN for VS segmentation. As shown in our proposed method, concatenating our T1W+C, and T2W images as multiparametric input cropped to 128 × 108 × 60 mm produced results (dice coefficient of 0.90 ± 0.05) that are remarkably close to those obtained by Wang et al.

The anisotropic MR image volumes with lower through-plane resolution would cause discontinuous tumor contour at through-plane direction. The results obtained using the single-pathway model with a single kernel size (3 × 3 × 3) for encoding was influenced by features manifesting in the through-plane direction, the spatial resolution of which is far lower than that of in-plane features. As shown in Fig. 1B, the tumor contour delineated by the dual-pathway model (Fig. 1B, left) successfully detected the bottom region of tumor, which was missed by the single-pathway model (Fig. 1B, right).

This study used multiple parametric MR images prior to GKRS as inputs for CNN training, with the aim of capturing tumor regions presenting inhomogeneous intensity. We implemented an automated pre-processing pipeline for MR images in RAW format, to create images that are compatible with existing deep-learning models. We then applied a novel U-Net model to the task of segmenting VS using multi-parametric MR images of lesions with a solid region (high-intensity T1W+C) as well as a cystic region (high-intensity T2W) (Fig. 1C). To the best of our knowledge, this is the first example of an end-to-end deep-learning segmentation method using multi-parametric MR images. Testing was performed using 1290 follow-up multi-parametric MR images from 381 VS patients. Note that the tumors were also delineated manually at the time of GKRS as well as at the time of follow-up for use as ground truth results. The accuracy of the AI model exceeded 99% in measuring the volume of tumor, and descripting the direction of tumor progression, regression, or pseudo-regression after radiation treatment. In the future, the proposed AI scheme could be applied to longitudinal follow-up assessments following a variety of therapeutic interventions.

In our previous study, Yang et al.^5^ constructed a two-level machine-learning model to predict the occurrence of transient pseudoprogression after GKRS. The prediction of transient pseudoprogression achieved an accuracy of 85.0% based on another five radiomic features associated with the inhomogeneous hypointensity pattern of contrast enhancement and the variation of T2-weighted intensity. Similarly, Speckter et al.^10^ found significant correlation of volumetric changes with texture analysis parameters of T2WI MR images for progression, pseudo-progression and regression. Langenhuizen et al.^17^ also found the correlations of textures on MRI for treatment outcome, particularly for pseudo-progression. In the near future, the imaging features within the tumor can predict the treatment response, and accurate longitudinal volumetric measurements showed in this study will be more and more important.

In conclusion, this paper outlines a novel approach to the evaluation of treatment responses (i.e., volumetric measurement of tumors) using MR images along a GKRS timeline. Artificial intelligence based on a deep-learning model was applied to a longitudinal MR imaging dataset of VS. Our results provide sequential snapshots of dynamic processes indicative of GKRS effects.

### Study limitations

This study faced a number of limitations, including those inherent to retrospective data collection. Because the location and contrast intensity of VSs were mostly consistent, the precision of AI model can be higher than other tumor identities (e.g. meningioma), and the generalizing ability to other data still need to be validated using external data. Hence, the applicability of the proposed AI model to other brain tumors will need to be carefully verified. We were unable to overcome the partial volume effects due to the fact that the upper and lower cut of tumors were amplified, which led to overestimates of tumor volume. We opted to include T2W images to deal with instances of cystic VS and VS with loss of central enhancement; however, this slightly elevated the errors in cases of VS with homogenous contrast enhancement. Finally, the registration of T1W+C and T2W images inevitably leads to small errors, regardless of the software used. In most cases, error of 0.1 mm is generally considered acceptable; however, errors of even this small magnitude would be sufficient to undermine the performance of the proposed AI model.

## Methods

### Patient population

A total of 381 patients were recruited for analysis, the clinical details of which are summarized in Table 1. The median age was 53.9 years old, and most of the patients were female (57.3%). Most of the VS were on the right side (n = 209, 54.9%), and the median tumor volume was 2.05 ml, based on estimates from MR images. Most of the patients presented neurological deficits, including hearing impairment (74.8%), vestibular deficits (68.0%), facial palsy (12.6%), adjacent cranial neuropathy (12.1%), and signs of brainstem or cerebellum compression, including long-tract (11.5%), and cerebellar (11.0%). Few of patients who had no symptoms and signs (n = 30, 7.9%) were incidentally found, and underwent GKRS directly.

### Patient consent and institutional review

Totally 381 patients, who underwent VS radiosurgery between 1993 and 2017, extracted from GKRS database of Taipei Veteran General Hospital (TVGH) constituted the patient cohort of current study. Patients with age of < 20-year-old at GKRS were excluded from the study. All patients gave written informed consent for their radiosurgery. The institutional review board (IRB) of TVGH approved the current study with exempting consent form (IRB number: 2018-11-008AC). The current study was anonymized, and followed the requirements of ethical regulation of the country and TVGH research guidelines and regulation.

### Gamma Knife radiosurgery and follow-up strategy

Radiosurgery was performed using Leksell Gamma Unit Model B, C, or Perflexion (Elekta Instrument, Inc). The prescription dose was generally set at an isodose level of 50–60%, and the median margin dose was 12 Gy. After GKRS, all of the patients underwent MRI examinations at 6-month intervals. Estimates of tumor volume were obtained through the analysis of MR images^5,6^. MRI analysis was used to classify tumor responses to GKRS within three categories: (1) regressed (i.e., stable) tumor volume was defined as a residual tumor volume of less than 110% of the original volume; (2) increased tumor volume was defined as a residual tumor volume exceeding 110% of the original tumor volume at the time of treatment; (3) pseudo-progression was defined as a transient increase in tumor volume within 6–18 months after GKRS. These classifications require confirmation via meticulous analysis of the images and volumetric measurements at each follow-up time point.

### Volumetric measurement

In the current study, we developed an end-to-end deep-learning scheme with automated pre-processing pipeline to elucidate changes in tumor volume. We applied the proposed method to multiple parametric MR images (T1W+C, and T2W) obtained from a series of 381 VS patients (1290 MR examinations). Note that all images were acquired using consistent imaging acquisition protocols.

In the following, we detail the imaging procedure and outline the evaluation metrics. The automated volumetric measurement scheme was implemented in three steps: (1) automated pre-processing of parametric MR images acquired after GKRS; (2) automated prediction of the tumor mask using a novel dual-pathway model; (3) estimation of tumor volume based on the predicted tumor mask and voxel size in MR images.

### Proposed MR image acquisition and image pre-processing schemes

**Figure 4:**
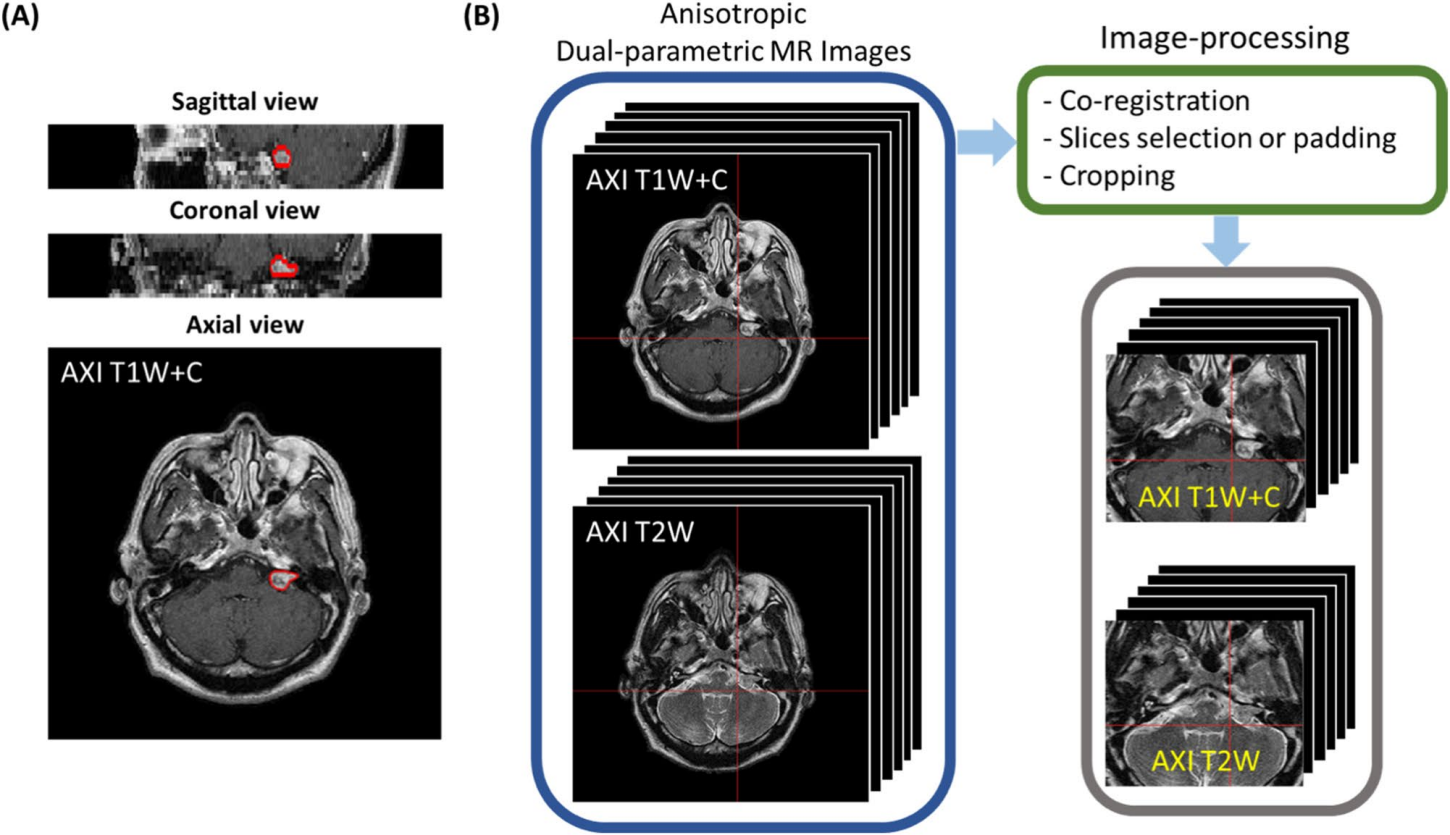
(**A**) Sagittal, coronal, and axial views of anisotropic MR images; (**B**) these raw MR images will be processed follow the steps showing here: co-registration between the various parametric images, and unified the image volume size before input our proposed dual-pathway U-Net model.

All stereotactic MR images were scanned using a GE scanner (1.5T). The MR images included (1) Axial 2D Spin Echo (TR = 416 ms, TE = 9 ms, flip angle = 90°) T1W+C images with a matrix size of 512 × 512 and voxel size 0.5 × 0.5 × 3 mm^3^ and (2) Axial 2D Spin Echo (TR = 4050 ms, TE = 109 ms, flip angle = 90°) T2W images with a matrix size of 512 × 512 and voxel size of 0.5 × 0.5 × 3 mm^3^ (Fig. 4a). Note that scanning a patient after GKRS could in no way guarantee that the acquisition position would match those of the stereotactic MR images used to train the deep-learning model. We sought to overcome some of the difficulties in applying follow-up MR images by constantly updating the training database with additional follow-up MR images.

We developed an automated image pre-processing module to ensure that all of the images used by the CNN were fixed in terms of size. T2W images were aligned to individual T1W+C images using a 12-parameter rigid-body registration algorithm in SPM12 (Statistic Parametric Mapping)^18^. We compensated for inter-subject variations in the number of slices by applying zero padding (cases of < 20 slices) or removing slices (cases of > 20 slices) to ensure that there were precisely 20 slices in each image volume. Otsu’s threshold^19^ was then used to generate a binary slice-wise image mask, which indicate the head region and also for the background noise suppression. The center of bounding box (256 × 216 px) was set at the center of head region to crop the MR images. Finally, the images were translated from 512 × 512 × *n* slices into 256 × 216 × 20 slices (Fig. 4b).

### Proposed end-to-end deep-learning scheme

**Figure 5:**
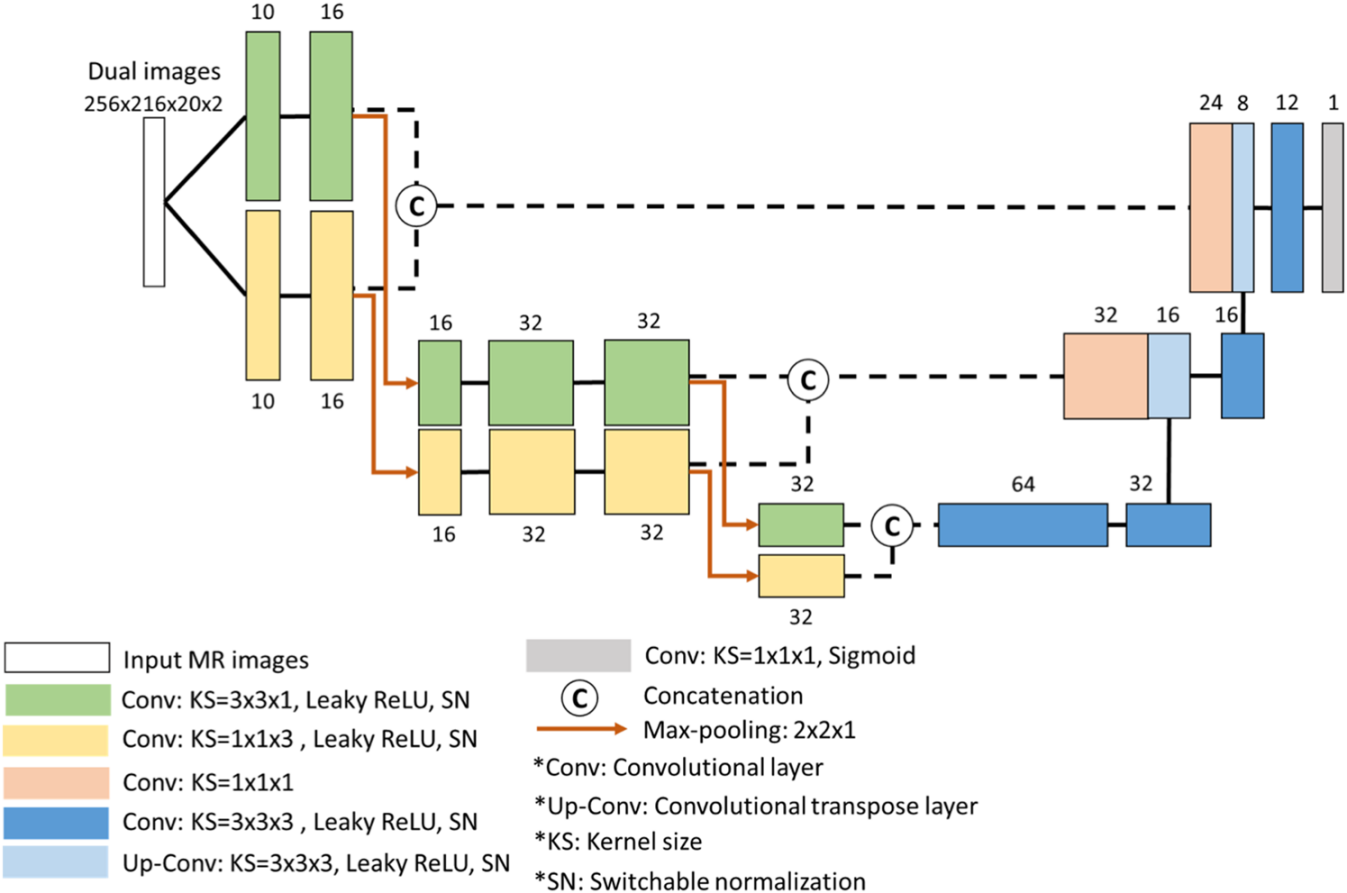
Architecture of proposed dual-pathway U-Net model for VS segmentation.

The proposed dual-pathway model was implemented as an encode–decode convolutional neural network (Fig. 5) inspiring by U-Net. The two convolution pathways in the encoder used convolution kernels of different sizes: 3 × 3 × 1 (for the extraction of in-plane features from anisotropic MR images) and 1 × 1 × 3 (for the extraction of through-plane features). Our dual-pathway U-Net model was trained using dice loss^20^ with additional L_2_ regularization. The dice loss was used to evaluate similarities in overlap between model predictions and the ground truth, whereas L_2_ regularization was used to prevent overfitting. We defined $x$ as the position of each voxel in the predicted mask $p\left(x\right)\in \left[\text{0,1}\right]$ or ground-truth mask $gt\left(x\right)\in \left\{\text{0,1}\right\}$. The weights in the CNN model were denoted by $W$, and ${\Vert W\Vert }_{2}$ was used as the *L*_2_ regularization term. The total number of training epochs was 60 with a batch size of 4. In the first 40 epochs, we used the Adam optimizer with a learning rate of 0.001. The loss function in the first 40 epochs was defined as follows:

1 $${L}_{DICE}=1-(2\sum p\left(x\right)\bigcap gt\left(x\right))/(\sum p\left(x\right)+\sum gt\left(x\right)+\epsilon ),$$

2 $${L}_{first40epochs}={L}_{DICE}+{\lambda }_{1}{\Vert W\Vert }_{2}.$$

We set $\epsilon =1\times {10}^{-5}$ as the smoothing term, and set ${\lambda }_{1}=1\times {10}^{-5}$ as the weight decay rate. In the last 20 epochs, we employed the Tversky index as a loss function^21,22^ and a stochastic gradient decent optimizer with a lower learning rate of 0.0001. We respectively defined $\overline{{p\left( x \right)}}$ and $\overline{{gt\left( x \right)}}$ as the complement of $p\left(x\right)$ and $gt\left(x\right)$ in order to calculate the incidence of false positives and false negatives in the prediction results. The Tversky loss function was defined as follows:

3 $${L}_{tversky}=1-\left(\sum p\left(x\right)\bigcap gt\left(x\right))/(\sum p\left(x\right)\bigcap gt\left(x\right)+\alpha \sum gt\left(x\right)\overline{{p\left( x \right)}}+\beta \sum \overline{{gt\left( x \right)}}p\left(x\right)+\epsilon \right), $$

4 $${L}_{last20epochs}={L}_{tversky}+{\lambda }_{1}{\Vert W\Vert }_{2}.$$

We used $\alpha$ and $\beta$ to adjust the weights of false positives and false negatives in the loss function. Initial trial-and-error analysis led us to set the two parameters respectively at 0.3 and 0.7.

The proposed dual-pathway model was trained using 416 MR image volumes from 416 patients (prior to GKRS), and 121 follow-up MR image volumes from 82 patients, and was tested via independent 100 image volumes from the other 100 patients (prior to GKRS). Each of training subject’s T1W+C and T2W image volumes were respectively normalized using z-score normalization, and concatenated together to serve as the multi-parametric input. The tumor masks that were used as training and testing ground truth were manually delineated by experienced neuroradiologists (coauthors including HM Wu, CC Wu, and WY Guo, specified in neuroradiology for at least 5 years) and neurosurgeon (conauthors including CC Lee, HC Yang, and WY Chung, specified in radiosurgery for at least 5 years) during treatment planning of GKRS or follow-up. For each MRI set, a neuroradiologist and a neurosurgeon would check the tumor delineation at the same time. The training process applied fourfold cross-validation.

For comparison, we also developed a single-pathway model with the same dual-pathway architecture but with all of the kernels (except those after concatenation) changed to 3 × 3 × 3. Note that the training data and training parameters were also the same ones used for the dual-pathway model.

The training platform and CNN model were implemented using Python 3.6, Tensorflow 1.15. The system ran on a PC with an i7-8700 CPU, 48 GB of RAM, and two 11-GB NVidia RTX 2080Ti GPUs.

### Volume estimation

After training and testing, the proposed model was used to segment out regions indicative of VS from 1290 follow-up MR image volumes. This made it possible to calculate the volume of the tumors using the predicted tumor mask and voxel size in the MR images. The total number of voxels *N(t)* predicted by the model was then multiplied by the voxel size (mm^3^) in the MR images to obtain the tumor volume (cm^3^), as follows:

5 $$Tumor \,volume \left({\text{mm}}^{3}\right)=[N(t)\times\, voxel\, size]/1000.$$

### Evaluation metrics

The performance of the models was evaluated using the dice coefficient^23^, which indicates the degree of similarity between model predictions P $\left(x\right)$ and the ground truth GT $\left(x\right)$. A dice coefficient of 1 indicates that the predicted value is identical to the ground truth, as shown below:

6 $$Dice=2\sum P\left(x\right)\bigcap GT\left(x\right)/\sum P\left(x\right)+\sum GT\left(x\right).$$

In order to compare the performance of clinically-measured tumor volume (CTV) with the tumor volumes measured by our proposed model (MTV), we adopted relative volume difference (RVD), defined in the following, as the evaluation metric:

7 $$RVD=\left(MTV-CTV\right)/CTV.$$

## Data Availability

All data generated or analysed during this study are included in this published article (and its Supplementary Information files).

## Code availability

Source codes of the study are published in GitHub with the following link: https://github.com/KenLee1996/Dual-pathway-CNN-for-VS-segmentation.
